# Chondroid tenosynovial giant cell tumour of the temporomandibular joint

**DOI:** 10.1093/jscr/rjab155

**Published:** 2021-04-24

**Authors:** Abdullah Kanbour, Michael Hurrell, Peter Ricciardo

**Affiliations:** Oral and Maxillofacial Surgery, Royal Perth Hospital, Perth, WA 6000, Australia; Oral and Maxillofacial Surgery, Royal Perth Hospital, Perth, WA 6000, Australia; Oral and Maxillofacial Surgery, Royal Perth Hospital, Perth, WA 6000, Australia

## Abstract

Chondroid tenosynovial giant cell tumour (TGCT) is an extremely rare disease affecting the temporomandibular joint (TMJ). This report details the peri-operative findings and treatment with custom TMJ replacement of an initially misdiagnosed chondroid TGCT involving the TMJ.

## INTRODUCTION

Tenosynovial giant cell tumour (TGCT) is a remarkably rare benign proliferative disorder of the synovium and can be categorized into localized-TGCT and diffuse-TGCT (D-TGCT) types [1]. The occurrence of D-TGCT, previously known as pigmented villonodular synovitis (PVNS), involving the temporomandibular joint (TMJ) is even rarer with <130 cases ever documented. Clinical diagnosis of D-TGCT is challenging, often resulting in misdiagnoses and diagnostic lags averaging 11.4 ± 12 months in delays [2]. Chondroid TGCT, also known as PVNS with chondroid metaplasia, is a distinct and even rarer subtype, which has a predilection for the TMJ [3]. To the authors’ knowledge, only 31 cases of chondroid TGCT involving the TMJ have ever been documented. We present an additional case of chondroid TGCT of the TMJ, with an initial misdiagnosis of synovial chondromatosis, and discuss the treatment modality involving tumour resection and custom total joint replacement.

## CASE REPORT

**Figure 1:**
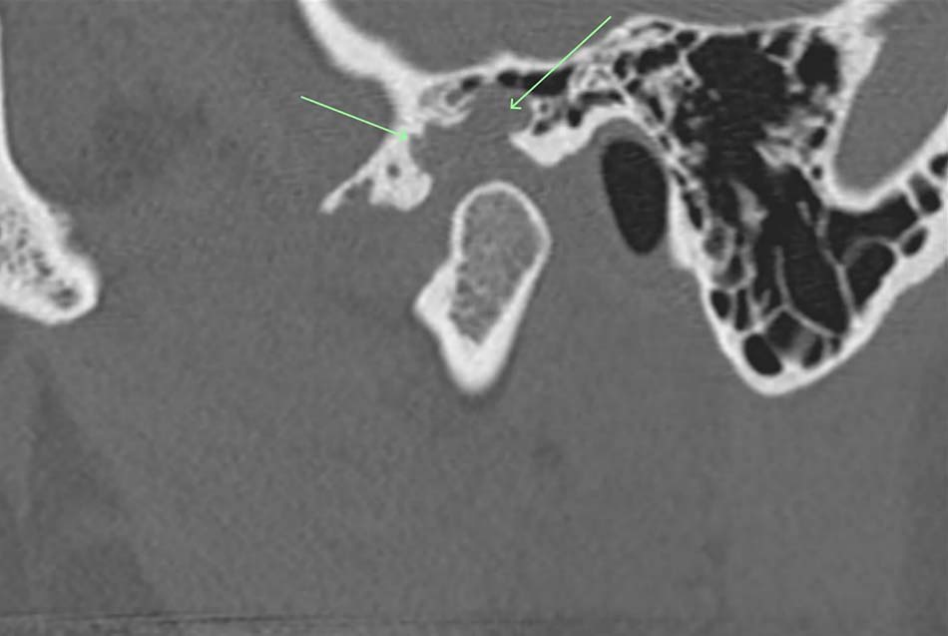
Non-contrast CT TMJs. Sagittal view showing the left TMJ erosive lesion within the glenoid fossa and articular eminence (green arrow).

**Figure 2:**
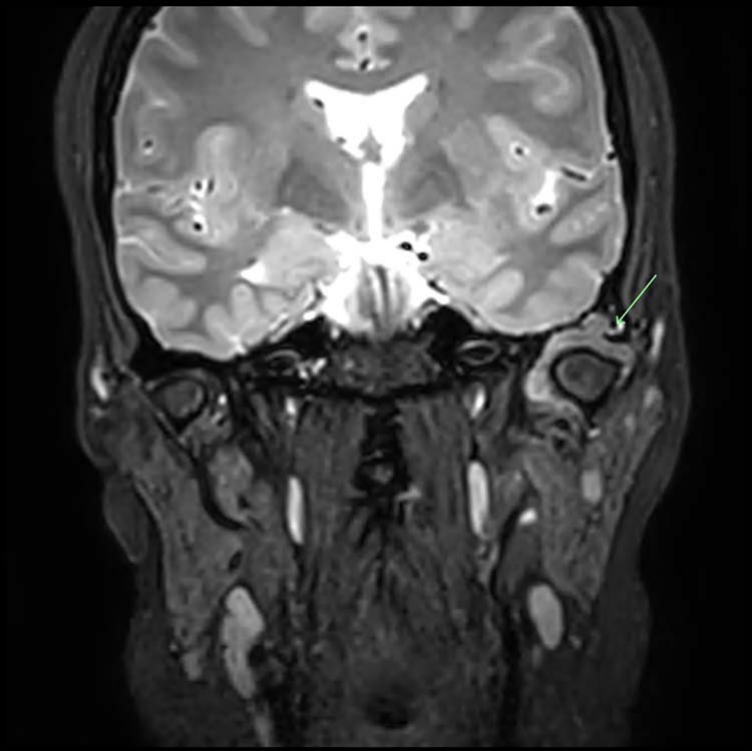
Post-gadolinium T2 MRI TMJs. Coronal view showing marked distension of the superior joint compartment with extensive scalloped lobulated erosion of the left temporal bone (green arrow).

A 33-year-old woman complained of a 3-month history of left-sided TMJ morning stiffness. She had never been treated nor undergone any previous TMJ procedures and reported an unremarkable medical history. Examination findings showed a small left-sided preauricular swelling with tenderness worse at the joint level. Mouth opening restricted by pain was 35 mm. Normal excursive movements and no malocclusion or lateral deviation was observed. Computed tomography (CT) and magnetic resonance imaging (MRI) of the TMJs showed: superior joint space effusion, multiple small low-signal intensity foci within the fluid and extensive erosions within the glenoid fossa, articular eminence and root of the zygoma (Figs 1 and 2). This was reported to be highly suggestive of left-sided TMJ synovial chondromatosis.

Arthroplasty (capsulectomy and discectomy) of the left TMJ with abdominal fat graft was performed. Capsule and soft tissue lesions extending into the bone were curetted and sent for histopathological analysis, which showed multinucleated giant cells and nodular immature chondroid metaplasia deemed to be a form of synovial chondromatosis.

**Figure 3:**
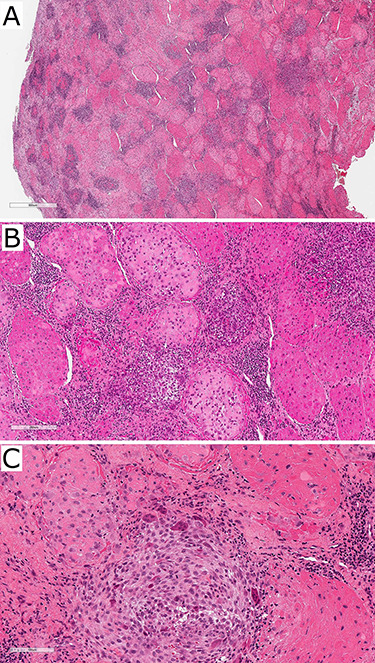
Photographs of haematoxylin and eosin-stained slides of the resected tissue sample showing fibrohistioctyic areas in addition to cartilaginous nodules. Magnification (**A**) ×94, (**B**) ×375, (**C**) ×750.

The patient was regularly reviewed reporting no improvement to stiffness on mouth opening. At her 3-month post-operative review, she complained of worsening stiffness to mouth opening. A follow-up CT of the TMJs portrayed extensive progressive bony erosion to the mandibular condylar head. This provoked a discussion with the pathologist to reconsider the diagnosis of the previously sent specimen. A histopathological revision reported a chondroid fibrohistioctyic lesion with overall features more in keeping with PVNS with chondroid metaplasia (Fig. 3).

**Figure 4:**
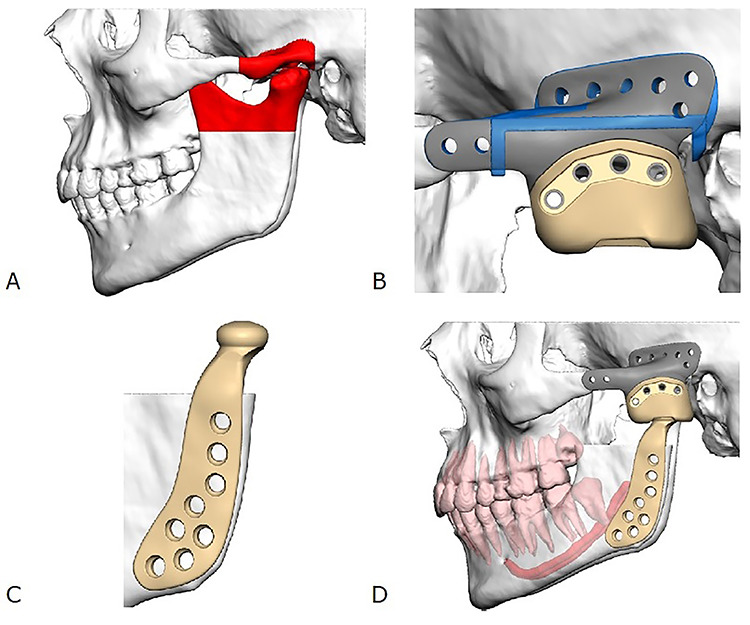
Illustration of the custom TMJ prosthesis. (**A**) Area planned for resection (red), (**B**) titanium zygomatic fossa component (grey) fixated to an UHMWPE fossa component (yellow), (**C**) titanium alloy mandibular component and (**D**) final relationship of all three components comprising the custom TMJ prosthesis.

The patient was subsequently planned for a second operation for resection of the affected tissue. To achieve clear surgical margins, this included the total resection of the left TMJ and the entire root of the zygoma along with the glenoid fossa. The TMJ was planned to be reconstructed with a custom TMJ prosthesis. Consequently, a three-component custom TMJ prosthesis (Zimmer Biomet) was fabricated. This included: (i) a titanium zygomatic root component to be fixated to the zygomatic arch and temporal bone, (ii) an ultra-high molecular weight polyethylene (UHMWPE) fossa component to be fixated into the zygomatic root component and (iii) a titanium (Ti-6Al-4V) alloy with titanium alloy coating mandibular component to replace the articular surface of the mandibular condyle (Fig. 4). Ordering and delivery of the custom TMJ prosthesis resulted in further treatment delay. The surgical procedure was conducted as planned. Histopathological findings of the resected specimen was consistent with the previously diagnosed PVNS with chondroid metaplasia with negative margins. The patient’s recovery was uneventful. She reported a full resolution of morning stiffness at 3-month post-operative review.

## DISCUSSION

D-TGCT is an articular pathology originating from a tendon sheath, joint capsule or bursae. The aetiology of this locally aggressive and proliferative tumour involves both inflammatory and neoplastic processes. This rare disease has an annual incidence of 1.8:1000 000 and predominantly affects the knees and hips of patients in their second to fourth decade of life with female predominance [4]. D-TGCT affecting the TMJ is even rarer with <130 cases ever documented. D-TGCT of the TMJ often presents with bony destruction of the mandibular condyle. Intracranial extension with skull base erosion is less common [1]. D-TGCT symptoms include: preauricular pain(less) mass, otalgia, tinnitus, hearing loss, trismus and TMJ crepitations. Headache, nausea and vomiting may occur if intracranial extension with intraparenchymal invasion is involved [3]. A differential diagnosis of a preauricular mass is extensive; however, D-TGCT of the TMJ is often overlooked and is featured on a differential list only 13% of the time prior to treatment [5].

Chondroid TGCT can mimic other cartilage-forming pathologies such as chondrosarcoma and synovial chondromatosis rendering its diagnostic challenge even greater [3, 6]. Between CT and MRI, chondroid TGCT manifests as an erosive bony lesion with its extent reliably identified. Focal hypointense areas on MRI are characteristic features secondary to the blooming artefact caused by the haemosiderin in the lesion [7, 8]. Definitive diagnosis is made histopathologically typically identifying fibrohistioctyic lesions comprised of plump histiocytoid cells and multinucleated giant cells, cartilaginous nodules and haemosiderin deposition [6]. Varying degrees of chondroid metaplasia is seen in the chondroid subtype [3]. Due to the aggressive locally destructive nature of the disease, D-TGCT treatment necessitates surgical resection of all affected tissue with clear margins. Reconstructions vary from bone grafts, total joint replacement to free vascular graft. Adjuvant radiotherapy may be utilized with extensive disease in difficult attainable sites or residual disease [8]. Recurrence of D-TGCT of the TMJ (9%) is lower than in other joints (8–46%); however, the accuracy of this comparison remains unclear given the rarity of this disease involving the TMJ and lack of longitudinal data [4].

Nevertheless, chondroid TGCT is a rare disease of the TMJ, which poses diagnostic delays and uncertainty. Our case demonstrated a definitive diagnosis and treatment were reached 8 and 18 months, respectively, following the patient’s initial presentation, in keeping with the delays described in the literature. The authors believe it is advisable to consider D-TGCT of the TMJ on the differential list of preauricular swellings, and treatment with complete resection, and close follow-up is paramount. The authors have demonstrated a custom TMJ prosthesis extending to local extra-articular surfaces is a viable solution for TMJ reconstruction in instances with extra-articular disease spread.
